# ‘I am afraid you will see the stain on my soul’: Direct gaze neural processing in individuals with PTSD after moral injury recall

**DOI:** 10.1093/scan/nsad053

**Published:** 2023-10-20

**Authors:** Krysta Andrews, Chantelle S Lloyd, Maria Densmore, Breanne E Kearney, Sherain Harricharan, Margaret C McKinnon, Jean Théberge, Rakesh Jetly, Ruth A Lanius

**Affiliations:** Department of Psychiatry and Behavioural Neurosciences, McMaster University, Hamilton, ON L8N 3K7, Canada; Homewood Research Institute, Guelph, ON N1E 6K9, Canada; Homewood Research Institute, Guelph, ON N1E 6K9, Canada; Department of Psychiatry, Western University, London, ON N6C 0A7, Canada; Department of Psychology, Neuroscience, and Behaviour, McMaster University, Hamilton, ON L8S 4K1, Canada; Department of Psychiatry, Western University, London, ON N6C 0A7, Canada; Imaging Division, Lawson Health Research Institute, London, ON N6A 4V2, Canada; Department of Neuroscience, Western University, London, ON N6A 3K7, Canada; Department of Psychiatry and Behavioural Neurosciences, McMaster University, Hamilton, ON L8N 3K7, Canada; Department of Psychiatry and Behavioural Neurosciences, McMaster University, Hamilton, ON L8N 3K7, Canada; Homewood Research Institute, Guelph, ON N1E 6K9, Canada; Mood Disorders Program, St. Joseph’s Healthcare Hamilton, Hamilton, ON L8N 3K7, Canada; Department of Psychiatry, Western University, London, ON N6C 0A7, Canada; Imaging Division, Lawson Health Research Institute, London, ON N6A 4V2, Canada; Department of Medical Biophysics, Western University, London, ON N6A 5C1, Canada; Canadian Forces, Health Services, Ottawa, ON K1A 0S2, Canada; Department of Psychiatry, University of Ottawa, Ottawa, ON K1N 6N5, Canada; Homewood Research Institute, Guelph, ON N1E 6K9, Canada; Department of Psychiatry, Western University, London, ON N6C 0A7, Canada; Imaging Division, Lawson Health Research Institute, London, ON N6A 4V2, Canada; Department of Neuroscience, Western University, London, ON N6A 3K7, Canada

**Keywords:** post-traumatic stress disorder, direct gaze, moral injury, fMRI

## Abstract

Direct eye contact is essential to understanding others’ thoughts and feelings in social interactions. However, those with post-traumatic stress disorder (PTSD) and exposure to moral injury (MI) may exhibit altered theory-of-mind (ToM)/mentalizing processes and experience shame which precludes one’s capacity for direct eye contact. We investigated blood oxygenation level-dependent (BOLD) responses associated with direct *vs* averted gaze using a virtual reality paradigm in individuals with PTSD (*n *= 28) relative to healthy controls (*n *= 18) following recall of a MI *vs* a neutral memory. Associations between BOLD responses and clinical symptomatology were also assessed. After MI recall, individuals with PTSD showed greater activation in the right temporoparietal junction as compared to controls (*T* = 4.83; *p*FDR < 0.001; *k* = 237) during direct gaze. No significant activation occurred during direct gaze after neutral memory recall. Further, a significant positive correlation was found between feelings of distress and right medial superior frontal gyrus activation in individuals with PTSD (*T* = 5.03; *p*FDR = 0.049; *k* = 123). These findings suggest that direct gaze after MI recall prompts compensatory ToM/mentalizing processing. Implications for future interventions aimed at mitigating the effects of PTSD on social functioning are discussed.

Human eye contact provides a rich source of social and non-verbal communication, as it is integral to creating bonds with others. Specifically, direct eye gaze is distinguished from averted eye gaze in that it signals one’s motivation to approach another (Ziaei *et al.*, 2017) to, in turn, facilitate social cognitive processes known as ‘theory of mind’ (ToM) or ‘mentalizing’ (Conty *et al.*, 2007). While mentalization helps individuals understand affective emotional states, ToM involves understanding the intentions and beliefs of others to navigate social interactions and engage in goal-directed behaviours (Frith and Frith, 2005). However, individuals with post-traumatic stress disorder (PTSD) often experience hypervigilance and alterations in arousal, which may create a bias toward perceived threat during direct eye contact (Krill and McKinnon, 2010; Wilkinson, 2010; Steuwe *et al.*, 2014) and diminish capacities to engage in social situations.

## The effect of PTSD and moral injury on direct eye gaze

PTSD develops after experiencing a traumatic event(s) and is characterized by a sequela of symptoms (e.g. intrusive memories, hypervigilance) (American Psychiatric Association, 2013). Traumatogenic symptomatology can also include pervasive feelings of shame and guilt, particularly for those who experience interpersonal trauma (Plante *et al.*, 2022), which significantly hinder relationships (Budden, 2009; Dorahy, 2010; Dorahy *et al.*, 2013; Cunningham, 2020) and may propagate feelings of isolation and disconnectedness from others (Lanius *et al.*, 2011). Thus, the threat imposed by direct eye gaze can physically and emotionally dysregulate individuals with PTSD, disrupting their understanding of the emotions, intentions, and behaviours of others. Indeed, deficits have been reported in tasks measuring ToM/mentalizing performance for individuals with PTSD (Nazarov *et al.*, 2014; McKinnon *et al.*, 2016).

Deficits in social cognitive processing linked to direct eye gaze may be particularly relevant for those whose traumatic experience stems from a moral injury (MI) that was interpersonal in nature. MI refers to the experience of either perpetration of a moral violation and/or perceived moral betrayal by a trusted individual or organization in power (Shay, 2014; Schorr *et al.*, 2018). Some examples include: experiences of abuse and/or neglect in childhood from a caregiver or trusted adult; being unable to help someone who is suffering (e.g. paramedics, firefighters); witnessing or killing another person in one’s military role; or not receiving necessary support for mental health difficulties acquired from one’s occupation. MI can foster a loss of confidence in one’s own or others’ ability to engage in ethical behaviours (Drescher *et al.*, 2011) and is linked to general decline in positive affect and resilience (Wisco *et al.*, 2017; Williamson *et al.*, 2018; Griffin *et al.*, 2019) and feelings of shame, rage and guilt (Litz *et al.*, 2009; Dombo *et al.*, 2013; Kopacz *et al.*, 2015; McEwen *et al.*, 2020; Brémault-Phillips *et al.*, 2022) that may become particularly salient when experiencing direct eye contact. One client described the experience as: ‘If somebody saw into my eyes and saw what I took part in….It feels, really scary. I feel like they’re going to see a kind of stain on my soul’ (Frewen and Lanius, 2015). Thus, direct eye contact becomes a vulnerable experience where the actions or inactions of traumatic experiences seem transparent to any person the individual encounters, suggesting that the experience of MI and associated feelings of shame can negatively distort how one views oneself in the world. Nevertheless, there is a dearth of research examining the associations between PTSD, MI and direct eye gaze. To our knowledge, no study has examined neural mechanisms of eye contact following a morally injurious event in individuals with interpersonal trauma-related PTSD.

## Neural processing of direct eye gaze in healthy individuals

The core neural regions involved in social cognition in healthy individuals include specialized perceptual processing areas such as the extrastriate cortex (‘body area’) involved in the perception of biological or intentional motion and the fusiform gyrus (‘face area’) involved in perceiving facial expressions (Stevens and Jovanovic, 2018). Additionally, the dorsomedial prefrontal cortex (DMPFC) has been implicated in self-referential processing (Saxe *et al.*, 2006; Schmitz and Johnson, 2007; Legrand and Ruby, 2009), which refers to how individuals relate to stimuli encountered in their environment (Northoff *et al.*, 2006). The DMPFC works in tandem with the temporoparietal junction (TPJ) and the bilateral temporal poles to carry out neural processes integral to ToM/mentalizing such as recognizing emotional facial expressions, adopting the perspective of others and understanding the motivations, behaviours and thoughts of others (Van Overwalle, 2009; Carter and Huettel, 2013; Schurz *et al.*, 2014). In particular, the TPJ is involved in processing transient social information whereas the DMPFC is involved in processing stable traits in others (Van Overwalle, 2009; Schurz *et al.*, 2014). Furthermore, when processing the emotional states of others, the bilateral insula and the mid-cingulate cortex are involved in passive observation and active evaluation, respectively (Fan *et al.*, 2011). The cerebellum has also been found to be significantly linked to ToM/mentalizing processes, such as ‘person mentalizing’ (e.g. ability to understand the traits, preferences and characteristics of people or oneself), ‘event mentalizing’ (e.g. understanding the intentions and beliefs behind the behaviours of others) and ‘abstract mentalizing’ (e.g. projecting oneself into the future and autobiographical recall) (Van Overwalle *et al.*, 2014). While some activity supporting these processes has been reported in the right anterior lobules IV and VI, most mentalizing processing occurs in the posterior regions of the cerebellum (e.g. bilateral Crus I/II) (Van Overwalle *et al.*, 2020). Indeed, a recent meta-analysis reported that 74% of the included studies showed significant associations between the posterior Crus II region and mentalizing and self-related emotional processing (Van Overwalle *et al.*, 2020). Taken together, the cerebellum may also support the social cognitive processes involved in direct *vs* averted eye gaze.

## Neural processing of direct eye gaze in individuals with PTSD

Compared to healthy controls, individuals with PTSD exhibit abnormal neural patterns during social cognitive processing (Frewen *et al.*, 2010; Lanius *et al.*, 2011; Steuwe *et al.*, 2014; Thome *et al.*, 2014; Rabellino *et al.*, 2018). However, few studies have explicitly compared the neural correlates of direct *vs* averted eye gaze in individuals with PTSD *vs* healthy controls. Some have postulated that, in healthy individuals, direct eye gaze activates higher cortical structures supporting social cognitive processing such as the superior temporal sulcus, medial prefrontal and orbitofrontal cortices and fusiform gyrus (Liddell *et al.*, 2004; Johnson, 2005) via pathways emanating from subcortical structures including the superior colliculus, periaqueductal grey, amygdala and thalamic pulvinar nuclei (Senju and Johnson, 2009; de Gelder *et al.*, 2011). Steuwe *et al.* (2014) examined blood oxygenation level-dependent (BOLD) responses in female participants exposed to a male avatar displaying direct *vs* averted eye gaze and found that healthy individuals showed a response in the DMPFC, left TPJ and right temporal pole during direct eye gaze. However, PTSD participants demonstrated increased BOLD responses in subcortical structures such as the superior colliculus and the periaqueductal grey which, in addition to the locus coeruleus, amygdala and post-central gyrus, comprise the ‘innate alarm system’ (Liddell *et al.*, 2005; Lanius *et al.*, 2017). Activation of this system supports reflexive defensive responses to threat (Steuwe *et al.*, 2014), and its chronic activation can limit higher-level cortical engagement of ToM processes (Thome *et al.*, 2014). Another study examining the salience network during direct eye gaze in individuals with PTSD found increased within-network functional connectivity with the left amygdala and right insula for participants with PTSD as compared to healthy controls (Thome *et al.*, 2014). While the salience network supports detection of and attention to external stimuli to inform behaviours aimed at maintaining cognitive, emotional and behavioural homeostasis (Craig, 2002; Seeley *et al.*, 2007; Menon and Uddin, 2010), it can also contribute to PTSD symptoms such as hypervigilance (Hayes *et al.*, 2012). Taken together, previous studies examining neural correlates of direct *vs* averted eye gaze support the possibility that direct eye gaze initiates activation of automatic, subcortical processes which may then modulate higher-order ToM/mentalizing processes.

As an extension of Terpou *et al.* (2022), we examined the neural correlates of direct eye gaze in civilians with interpersonal trauma-related PTSD as compared to healthy controls following recall of a morally injurious *vs* neutral memory during a virtual reality paradigm based on previous studies (e.g. Schrammel *et al.*, 2009; Steuwe *et al.*, 2014). Notably, we manipulated gaze interaction (i.e. direct *vs* averted), not emotional expression, based on the findings by Steuwe *et al.* (2014) that group differences were independent of the emotions displayed by facial expressions. We hypothesized that the PTSD group would show increased activation in the subcortical structures involved in the innate alarm system (e.g. superior colliculus, locus coeruleus, amygdala) and lower activation in cerebellar and higher-order cortical structures involved in ToM and self-referential processes (e.g. TPJ, DMPFC, bilateral temporal poles) (Liddell *et al.*, 2004; Senju and Johnson, 2009; de Gelder *et al.*, 2011; Van Overwalle *et al.*, 2014).

## Methods

### Participants

Participants were recruited via advertisements within local mental health treatment centres and the community. The final sample consisted of forty-six participants including those with a primary PTSD diagnosis related to civilian trauma (*N *= 28) and MI event-exposed, healthy controls (*N *= 18). The group with a PTSD diagnosis was comprised of individuals with occupational or interpersonal-related PTSD as an extension of the analyses reported in Terpou *et al.* (2022). Demographics and clinical characteristics of the sample are presented in Table 1.

**Table 1.** Sample demographic and clinical information

	PTSD group	Control group
*N*	28	18
Sex	25 males, 3 females	7 males, 11 females
Age	48.5 ± 8.3	33.1 ± 10.9
CAPS-5 Total	41.7 ± 6.7**	0.2 ± 0.7
CAPS-5 B	11.7 ± 3.0**	0.1 ± 0.3
CAPS-5 C	4.5 ± 1.1**	0.0 ± 0.0
CAPS-5 D	15.4 ± 3.2**	0.1 ± 0.3
CAPS-5 E	10.1 ± 2.3**	0.0 ± 0.0
MDI	68.0 ± 18.2**	36.6 ± 6.3
CTQ	78.7 ± 25.7**	36.1 ± 10.2
IRI	65.0 ± 13.9	60.9 ± 13.2
IRI-PD subscale	12.0 ± 5.6*	7.5 ± 6.0
TRSI	62.7 ± 17.4**	25.6 ± 2.8
BDI	33.8 ± 10.4**	3.6 ± 4.2
MIES	38.5 ± 8.2**	27.4 ± 9.0
Psychotropic medication	23	0

* *P *< 0.05, **P < 0.01. Asterisks indicate significantly higher clinical symptom values relative to the control group.

Abbreviations: CAPS-5 = Clinician-Administered PTSD Scale for DSM-5; CAPS-5 B subscale = Re-experiencing symptoms; CAPS-5 C subscale = Avoidance symptoms; CAPS-5 D subscale = Negative alterations in cognitions and mood; CAPS-5 E subscale = Alterations in arousal and reactivity; MDI = Multiscale Dissociation Inventory; CTQ = Childhood Trauma Questionnaire; IRI = Interpersonal Reactivity Index; PD = personal distress subscale; TRSI = Trauma-Related Shame Inventory; BDI = Beck Depression Inventory; MIES = Moral Injury Events Scale.

Exclusion criteria included a lifetime diagnosis of bipolar or psychotic disorder, alcohol or substance use disorder in the three months prior to study participation, noncompliance with 3 Tesla functional magnetic resonance imaging (fMRI) safety standards, pregnancy, head injury with loss of consciousness, a neurological or pervasive developmental disorder and significant untreated medical illness. Additionally, control participants were excluded if they had any history of psychiatric disorders and were screened using the Moral Injury Events Scale (MIES; Nash *et al.*, 2013) to confirm exposure to potentially morally injurious events.

Approval was obtained for the study protocol via the Health Sciences Research Ethics Boards at Western University (#107575). Participants provided informed written consent prior to study involvement and were compensated $25 for the assessment interview and $50 for the MRI scanning visit. Participants recruited out of town were also reimbursed for travel expenses to London (e.g. mileage and parking).

### Clinical interviews

Participants completed the Structured Clinical Interview for DSM-IV Axis-I Disorders—Research Version (First *et al.*, 2002) to determine psychiatric history for study inclusion and determine comorbidities. The Clinician-Administered PTSD Scale for DSM-5 (CAPS-5) (Weathers *et al.*, 2013) was administered to evaluate PTSD symptom severity. Dissociative symptoms were assessed via the Multiscale Dissociation Inventory (MDI) (Briere *et al.*, 2005) and child maltreatment and neglect were evaluated using the Childhood Trauma Questionnaire (CTQ) (Bernstein *et al.*, 1994). Participants also completed the Interpersonal Reactivity Index (IRI) (Davis, 1980). The IRI evaluates perspective taking (i.e. ability to adopt the psychological point of view of others spontaneously), fantasy (i.e. ability to imaginatively experience the feelings and actions of fictitious characters in plays, books etc.), empathic concern (i.e. feelings of concern and sympathy for others) and personal distress (i.e. feelings of anxiety and unease in stressful interpersonal contexts). The Beck Depression Inventory-II (Beck *et al.*, 1996) was administered to assess attitudes and symptoms of depression during the previous two weeks. Finally, participants also completed the 24-item Trauma-Related Shame Inventory (TRSI), which assesses internalized and externalized experiences of shame that are attributed to trauma (Oktedalen *et al.*, 2014).

During the clinical assessment, participants were asked to describe a MI and neutral event that occurred within the same time period in eight sentences. They were provided with multiple MI definitions and discussed further with research staff to ensure the chosen events were appropriate (see supplementary materials for specific instructions) and reflective of 5–8/10 subjective units of distress to match distress intensity across groups. Following the procedure consistent with the Autobiographical Interview Administration Manual (Levine *et al.*, 2002; McKinnon *et al.*, 2015), recall was facilitated in a step-wise manner by reading and presenting the eight sentences in chronological order to the participant in the scanner. Self-report ratings of experienced shame, guilt and betrayal intensities as well as items related to dissociation and re-experiencing from the Responses to Script-Driven Imagery Scale (RSDI; Hopper *et al.*, 2007) were collected after each script (See Table 2).

**Table 2.** Participant responses to the Script-Driven Imagery Scale after the moral injury and neutral conditions

	PTSD group						Control group					
	Shame		Guilt		Betrayal		Shame		Guilt		Betrayal	
	Neutral	MI	Neutral	MI	Neutral	MI	Neutral	MI	Neutral	MI	Neutral	MI
Not at all (%)	85.7	7.1	82.1	10.7	78.6	14.3	88.9	18.8	93.8	12.5	100	50.0
Somewhat (%)	14.3	21.4	17.9	3.6	14.3	7.1	0	43.8	6.3	25	0	37.5
Moderately so (%)	0	17.9	0	21.4	3.6	7.1	0	31.3	0	37.5	0	12.5
Very much so (%)	0	53.6	0	64.3	3.6	71.4	0	6.3	0	25	0	0
Mean (SD)	1.1 (0.4)	3.2 (1.0)	1.2 (0.4)	3.4 (1.0)	1.3 (0.7)	3.4 (1.1)	1 (0)	2.3 (0.9)	1.1 (0.3)	2.8 (1.0)	1.00 (0)	1.6 (0.7)

Note. Participants completed the Responses to Script-Driven Imagery Scale (RSDI) after each condition (neutral, moral injury (MI)) and rated how much they were experiencing feelings of shame, guilt and betrayal from 1 (Not at all) to 4 (Very much so).

### Experimental setup

**Fig. 1.**
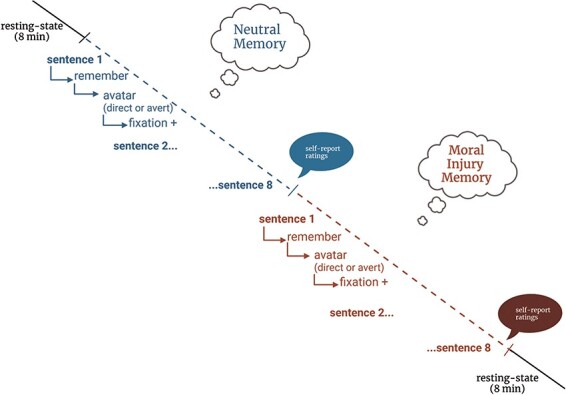
Experimental paradigm.

Participants lying supine on the MR scanner bed were presented with their neutral memory script followed by their MI memory script with a 2-min break in between according to standard methods (Lloyd *et al.*, 2021). The experimental paradigm was preceded and followed with resting-state scans of eight-minute duration for the purpose of future analyses. For each script, sentences were presented one by one. The first sentence was presented visually and simultaneously read aloud to the participant via MR compatible headphones in a neutral affective tone for 5 s. Next, participants spent 25 s recalling that part of their memory (detailed protocol is published elsewhere (Lloyd *et al.*, 2021)). Following recall, the avatar protocol was presented and then the procedure was repeated for the next sentence (see Figure 1 below). E‐Prime 3.0 software (Psychology Software Tools, Pittsburgh, PA) was used to present stimuli. An MR-compatible button press was used by participants after each script to rate the degree to which each memory induced (state) shame (1 = not at all and 4 = very much so).

### Gaze paradigm: avatar protocol

**Fig. 2.**
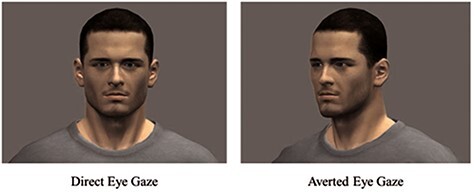
Avatar direct and averted eye gaze presented to the participant on the screen in the scanner following moral and neutral memory recall.

Consistent with the stimuli and paradigm outlined by Schrammel *et al.* (2009), after the MI or the neutral event recall, video sequences were presented that involved an avatar moving across the screen and either turning directly to the participant or at a 30° left or right angle to indicate that the avatar may be looking at something or someone else (See Figure 2). Only male avatars were used as they received higher ratings on naturalness, dominance and sociability as per Schrammel *et al.* (2009). Each video sequence involved three epochs for a fixed interval of 8.93 s.

In the first epoch, the avatar enters the screen from the left or right (2.44 s). In the second epoch, the avatar then turns their head and body towards the participant indicative of mutual eye-to-eye contact (direct gaze) or at 30° (averted gaze) (4.05 s). In the final epoch, the avatar turned away and left the screen from the opposite side from where it entered (2.44 s). Eight 8.93 s video sequences were presented, each followed by a 5 s fixation cross. Immediately after the scanning session, participants completed the Tonic Immobility Scale (Forsyth *et al.*, 2000). They were also shown still pictures of the avatar facial expressions and asked to identify the emotion the avatar was expressing as well how they felt in response to the avatar (e.g. shame, helplessness, sadness) presented in Table 3.

**Table 3.** Means and standard deviations of participant ratings of their emotions in response to the avatars displaying neutral affect

	PTSD group						Control group					
Avatar	Shame	Guilt	Betrayal	Helpless	Anger	Sad	Shame	Guilt	Betrayal	Helpless	Anger	Sad
**1**	1.7 (1.7)	1.5 (1.4)	1.8 (2.2)	2.8 (3.0)	2.5 (2.6)	2.4 (2.4)	1.0 (0.0)	1.0 (0.0)	1.0 (0.0)	1.0 (0.0)	1.0 (0.0)	1.0 (0.0)
**2**	1.2 (0.5)	1.3 (0.7)	2.0 (2.2)	2.5 (2.6)	2.3 (2.5)	2.3 (2.3)	1.0 (0.0)	1.0 (0.0)	1.0 (0.0)	1.0 (0.0)	1.0 (0.0)	1.0 (0.0)
**3**	1.8 (1.9)	1.6 (1.5)	2.9 (2.6)	3.2 (2.9)	3.2 (2.9)	3.2 (2.8)	1.0 (0.0)	1.0 (0.0)	1.0 (0.0)	1.0 (0.0)	1.0 (0.0)	1.1 (0.2)
**4**	1.9 (1.8)	2.1 (2.2)	2.8 (2.6)	2.6 (2.3)	3.2 (2.8)	3.4 (3.1)	1.0 (0.0)	1.0 (0.0)	1.0 (0.0)	1.0 (0.0)	1.0 (0.0)	1.0 (0.0)
**5**	1.8 (1.8)	1.4 (1.1)	1.5 (1.6)	2.0 (2.1)	2.2 (2.4)	2.2 (2.2)	1.0 (0.0)	1.0 (0.0)	1.0 (0.0)	1.0 (0.0)	1.0 (0.0)	1.0 (0.0)
**6**	1.4 (1.1)	1.5 (1.2)	2.1 (2.4)	2.0 (2.2)	2.0 (2.0)	2.7 (2.9)	1.0 (0.0)	1.0 (0.0)	1.0 (0.0)	1.0 (0.0)	1.0 (0.0)	1.0 (0.0)
**7**	1.8 (2.1)	1.6 (1.5)	1.7 (1.8)	2.9 (2.5)	2.9 (2.9)	2.4 (2.2)	1.0 (0.0)	1.0 (0.0)	1.0 (0.0)	1.0 (0.0)	1.0 (0.0)	1.0 (0.0)
**8**	1.8 (1.5)	1.7 (1.3)	1.9 (1.8)	2.3 (2.2)	2.4 (2.6)	2.6 (2.2)	1.0 (0.0)	1.0 (0.0)	1.0 (0.0)	1.1 (0.2)	1.0 (0.0)	1.1 (0.2)

Note. Participants were shown images, one by one, of eight avatars with neutral facial expressions and asked to identify how they felt while watching the avatar in terms of shame, guilt, betrayal, helplessness, anger and sadness. Participants rated their feelings from 1 ([emotion] not present) to 9 ([emotion] extremely [present]). Avatar 1 = brunette, direct gaze; 2 = brunette, averted gaze; 3 = brunette, direct gaze; 4 = brunette, averted gaze; 5 = blonde, direct gaze; 6 = blonde, averted gaze; 7 = blonde, direct gaze; 8 = blonde, averted gaze.

### fMRI image acquisition

A 3 Tesla MRI Scanner (Biograph mMR; Siemens Medical Solutions) was used for brain imaging with a Siemens 32-channel head coil locally adapted to this scanner’s 4-plug interface. We collected orthogonal scout images, which were used to prescribe a tri-dimensional T1-weighted anatomical image of the whole head with 1 mm isotropic resolution (Magnetization Prepared Rapid Gradient Echo). Functional whole-brain images with BOLD contrast were acquired transversely with the manufacturer’s gradient echo, T2*-weighted blipped-echo-planar sequence (TE = 20 ms, TR = 3000 ms, FOV = 256 × 256 mm, flip angle = 90°, in-plane resolution = 2 × 2 mm), parallel imaging acceleration factor = 4). Each volume included 60 ascending interleaved slices with a thickness of 2 mm. We stabilized participants’ heads with foam padding, and the experimental runs each consisted of 118 volumes.

### fMRI pre-processing

Functional images were pre-processed using SPM12 (Welcome Trust Centre for Neuroimaging, London, UK: http://www.fil.ion.ucl.ac.uk/spm) within MATLAB R2019 (MathWorks Inc., MA). The pre-processing protocol involved discarding the four initial functional volumes, re-orientation to the anterior-posterior commissure axis, spatial alignment to the mean image using a rigid body transformation and re-slicing, and co-registration of the functional mean image to the subject’s anatomical image. Co-registered images were segmented using the ‘New Segment’ method in SPM12. Functional images were normalized to MNI space (Montréal Neurological Institute) and were smoothed with a full-width half maximum Gaussian kernel of 6 mm (Beissner *et al.*, 2011). Additional correction for motion was implemented using the Artifact Removal Tool (ART) software package (Gabrieli Lab, McGovern Institute for Brain Research), which computes regressors that account for outlier volumes.

### Statistical analyses

#### First-level analysis

All events (rest, instructions, fixation and conditions) were modelled as brain activation blocks combined with the hemodynamic response function for each participant. 117 volumes were used, with one additional volume excluded because presentation of stimuli ended partway through its acquisition. At this stage, functional data were high-pass filtered, and serial correlations were accounted for using an autoregressive AR (1) model; ART software regressors were included as nuisance variables to account for any additional movement artifacts. The direct and averted eye gaze experimental conditions were modelled separately on the first level for each participant.

#### Second-level analysis

Contrast images for direct and averted gaze were entered into second-level analyses in SPM12 to examine between group differences in neural activation during direct gaze processing. First, a full factorial split plot analysis of variance was conducted to explore the interaction between participant group (PTSD, control), and condition (direct, avert) via a 2 (group) x 2 (condition) matrix. Group differences in neural activation were examined at the whole brain level for the two gaze and MI conditions with separate two-sample t-tests. Moreover, a region of interest (ROI) analysis was conducted to examine cerebellum activation in both the PTSD and control groups. For our ROI, we selected the Spatially Unbiased Infratentorial Template Toolbox (SUIT) mask (Diedrichsen, 2006). The SUIT template mask includes a high-resolution, MNI-normalized cropped cerebellum and brainstem. Given the large size of the mask, it can be considered a conservative selection as an ROI. We used voxel-wise thresholds set to *P* < 0.001 uncorrected. For between-group analyses, a conservative significance threshold was set to *P* < 0.05 FDR-corrected for cluster-level significance. Neural structures were confirmed via visual inspection using the neuromorphometrics atlas in SPM, AAL atlas and cross-referencing MNI coordinates with Brodmann’s areas.

## Results

### Demographics and clinical measures

As expected, the PTSD group scored significantly higher for CAPS-5 total score (and subscales), MDI, CTQ, IRI-PD, TRSI, BDI and MIES as compared to the control group. Also, while we found a significant difference between groups for age, upon closer examination of the influence of age on the BOLD signal yielded non-significant differences across conditions. No significant differences were found between groups for sex.

### Full factorial design

#### Between-group

**Fig. 3.**
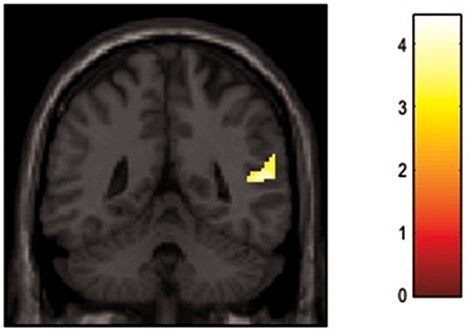
Activation in the right temporoparietal junction in the PTSD group as compared to the control group.

The whole brain analysis revealed a significant main effect of group (control, PTSD) (MNI = 52, −46, 12; *F* = 17.4; *Z* = 3.8; pFDR cluster corrected = 0.025; k = 84). In follow up *t*-tests, the contrast (Direct > Avert gaze, MI memory recall) showed greater activation of the right TPJ in the PTSD group as compared to the control group (MNI = 52, −46, 12; *T* = 4.83; *Z* = 4.5; pFDR cluster corrected < 0.001; k = 237; see Figure 3). Direct > Avert gaze during neutral memory recall did not yield any significant activation (see Figure S1 in supplementary material).

#### Within-group

When looking at direct *vs* averted eye gaze (direct > avert) in the PTSD group, significant activation was observed in the cerebral vermis 8 (MNI = − 2, −70, −38; *T* = 5.3; Z = 4.9; pFDR cluster corrected = 0.046; k = 93), the right superior temporal gyrus (MNI = 54, −44, 12; *T* = 5.0; *Z* = 4.7; pFDR cluster corrected = 0.003; k = 181) and the right angular gyrus (MNI = 50, −46, 34; *T* = 4.0; *Z* = 3.8; pFDR cluster corrected = 0.033; k = 108). See Figures S2-S4 in the supplementary materials. No significant within-group findings emerged for the control group.

### Clinical correlations

#### PTSD Group

**Fig. 4.**
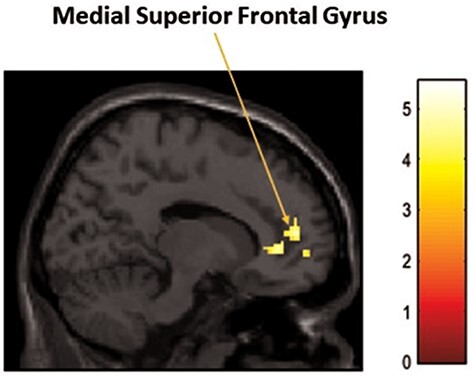
Significant positive correlation between scores of IRI-PD and activation in the right medial superior frontal gyrus in PTSD group.

**Fig. 5.**
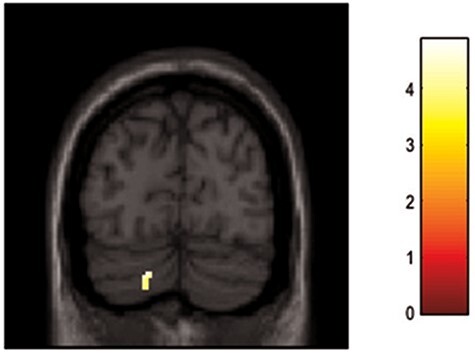
Significant negative correlation between scores of the TRSI and activation in the left cerebellum crus II in PTSD group via ROI analysis.

Follow-up clinical correlations were conducted in the PTSD group during direct *vs* averted eye gaze (direct > avert). Here, a significant positive correlation was observed between the IRI Personal Distress (IRI-PD) subscale and activity in the right medial superior frontal gyrus (MSFG), MNI = 14, 50, 12; *T* = 5.03; Z = 4.14; *p*FDR cluster-corrected = 0.049; k = 123 (see Figure 4). Further, an ROI analysis yielded a significant negative correlation between scores of the TRSI and the left cerebellum crus 2, MNI = − 16, −80, −32; *T* = 4.87; Z = 3.99; pFDR cluster corrected = 0.048; k = 37 (see Figure 5). No other clinical correlations yielded significant results (e.g. BDI, CTQ, CAPS-5 total and subscales).

## Discussion

The current study sought to identify the neural correlates of direct eye gaze (*vs* avert) in participants with PTSD as compared to healthy controls following recall of a MI or neutral memory. Our findings revealed that following recall of a morally injurious memory, but not a neutral memory, the PTSD group showed greater activation in the TPJ as compared to controls, an area central to multisensory processing and ToM/mentalizing processes (e.g. self-reflection, perspective taking) (Van Overwalle, 2009; Carter and Huettel, 2013; Schurz *et al.*, 2014). Further, those with PTSD demonstrated a significant positive association between right MSFG activation and experiences of personal distress in stressful interpersonal situations, as well as a negative association between cerebellum crus II activation and higher levels of trauma-related shame following recall of the MI memory. These findings may suggest that there are compensatory efforts by higher-order cortical structures to support one’s evaluation of the nature of the direct eye gaze following recall of a morally injurious event. Moreover, theorized top-down modulation may be enhanced when experiencing anxiety within the social context that direct eye gaze creates. Conversely, bottom-up ToM/mentalizing processes supported by the cerebellum may be disrupted by experiences of shame particularly within the context of recalling a MI. Taken together, these findings suggest an alteration in ToM/mentalizing processes of individuals with PTSD after MI, but not neutral, memory exposure during direct eye gaze processing.

### Neural correlates of direct gaze in the PTSD group

Increased activation of the TPJ in the PTSD group (as compared to controls) during direct gaze after recalling a MI, but not neutral memory, suggests that they may attempt to engage their altered ToM/mentalizing skills when faced with the direct gaze of the avatar. Notably, these findings were not aligned with the initial study hypothesis that direct eye gaze would show activation restricted to the automatic ‘innate alarm’ subcortical network in the PTSD group (e.g. superior colliculus/periaqueductal grey), which would limit engagement of higher-order cortical structures implicated in social cognitive processes (e.g. TPJ, medial prefrontal and orbitofrontal cortices) (Liddell *et al.*, 2004; Johnson, 2005; Senju and Johnson, 2009). These results contrast with the findings by (Steuwe, et al., 2014)of diminished ToM-related cortical engagement in PTSD during direct gaze.

One notable difference in the current study was our inclusion of a personalized MI-event script presented to participants prior to the avatar displaying direct or averted eye gaze. Participants chose events that would elicit a mid-to-high range moral emotion response (e.g. shame). It is possible that this methodological difference resulted in activation of the innate alarm system during MI recall which was then overcome by top-down modulation when the avatar was displayed. Consistent with this postulation, our colleagues found that during script-driven MI event-related memory recall, participants with PTSD showed increased activation both in subcortical areas of defensive responding (e.g. innate alarm system) *and* in cortical regions involved in modulation (e.g. dorsolateral prefrontal cortex) (Lloyd *et al.*, 2021).

Importantly, there were no significant differences between the PTSD and control groups during direct (*vs* avert) gaze when recalling a neutral event, which suggests that these neural response patterns may be unique to morally injurious events; similar findings exist in related studies (Lloyd *et al.*, 2021; Terpou *et al.*, 2022). Exposure to a MI can elicit emotions that may result in a wider distribution of activity across neural regions responsible for social cognition among others (e.g. moral reasoning, emotion regulation) (Bryan *et al.*, 2018). Though speculative, there may be competition in activation between the subcortical and cortical neural regions subsequent to the MI memory recall task when the participant is exposed to direct eye gaze (via the avatar). Furthermore, given that direct eye gaze can capture and hold attention (Senju and Hasegawa, 2005), the avatar may have required increased use of attentional processes that involve activation in higher-order cortical areas overlapping with those involved in ToM/mentalizing. As direct gaze increases autonomic arousal (Hietanen *et al.*, 2008; Helminen *et al.*, 2011), it may be possible that participants experienced heightened arousal corresponding with activation of the innate alarm system, but then suppressed it through heightened convergent activation in cortical structures from both attentional and ToM/mentalizing neural processes. Future research should consider measuring physiological arousal in addition to fMRI to determine whether individuals with MI-related PTSD suppress/over modulate the innate alarm system.

Interestingly, the use of the virtual characters—as opposed to a ‘live’ person—in the current study has been utilized previously (Schrammel *et al.*, 2009; Steuwe *et al.*, 2014; Thome *et al.*, 2014) and studies have demonstrated differences in neural activation in participants presented with a robot with a biological appearance (or virtual character) as compared to a robot without biological appearance or a human (Saygin *et al.*, 2011). It is possible that the participant perceives a virtual character as unpredictable, resulting in activation of subcortical structures to initiate a defensive response (e.g. innate alarm system) (Steuwe *et al.*, 2014). However, it is also possible that, while still threatening, the virtual character additionally elicits an evaluative, reflective response from participants given the knowledge that the character is not real. This supports the observed TPJ activation given its involvement in processing transient social cues. Future studies should aim to explore potential differences in the neural activation patterns of individuals with and without PTSD when presented with direct eye gaze from a virtual *vs* live person.

### Neural correlates of personal distress and trauma-related shame

Among individuals with PTSD exposed to a MI, direct eye contact was linked to greater activation in the right MSFG with increasing scores on the IRI-PD, which translate to higher anxiety and discomfort in social contexts. MSFG has been linked to self-reflective processes such as self-evaluation and self-judgment and processing negative self-related emotions, such as shame (Goldberg *et al.*, 2006; Thome *et al.*, 2014; Frewen *et al.*, 2020). Further, higher IRI-PD scores have been reported in other samples of individuals with PTSD (Parlar *et al.*, 2014) and can behaviourally manifest as fearfulness, social dysfunction and emotional vulnerability (Davis, 1983). Indeed, one client noted: ‘To make any kind of eye contact was to bring attention to myself, and it would be met 99% of the time with being slugged or hit in some way…I learned not to make eye contact, not to move, not to speak, and not to do anything. I learned: “keep your eyes down all the time”’ (Frewen and Lanius, 2015). Thus, those with traumatic and morally injurious experiences may avoid direct eye contact to defend against a perceived potential threat and subsequent emotional dysregulation. It is also possible that individuals with PTSD experiencing heightened anxiety can struggle to evaluate their role in a social interaction and process associated trauma-related emotions. The positive association between personal distress and MSFG activation may be indicative of an effort to compensate for a disruption in self-referential and emotional processing. Indeed, activity in the frontal cortex in response to eye contact aids in attentional and mentalizing processes to evaluate the other person’s intention and generate a planned behavioural response (Halgren and Marinkovic, 1995; Panksepp, 1998; Liddell *et al.*, 2004). The ambiguity of direct eye contact from a neutral expression on the avatars may exacerbate this compensatory effort given the amount of processing required to evaluate the context (Neta and Whalen, 2010). Indeed, individuals tend to perceive neutral stimuli negatively (Said *et al.*, 2011), and this may be especially true for individuals with PTSD who tend to have an enhanced sensitivity for detecting potential threat (Krill and McKinnon, 2010; Wilkinson, 2010; Aupperle *et al.*, 2012). Notably, this was partially captured in the PTSD group participants’ ratings of their emotions in response to the avatars. Participants in the control and PTSD groups were both shown still images of avatars with neutral facial expressions with direct and averted eye gaze and asked to rate how they felt from 1 ([emotion] not present) to 9 ([emotion] extremely [present]). For all of the possible feelings (i.e. shame, guilt, betrayal, helplessness, anger, sadness), the control group consistently reported not experiencing any of the emotions after presentation of each avatar (i.e. mean range = 1.00 to 1.06). However, the ratings were slightly more variable for the PTSD group with the highest mean rating reaching 3.40 (SD = 3.12) for sadness (see Table 3). This slight variability in the PTSD group ratings provides support for the possibility that the neutral expression of the avatar provided ambiguity that participants tended to perceive more negatively than the control group.

The ROI analysis also demonstrated significant associations between lower activation in the left cerebellum crus II and higher scores on the shame inventory suggesting that the experience of shame may disrupt crus II-mediated social cognitive processes. Indeed, childhood trauma, for example, is significantly linked to maladaptive self-processes including shame and self-criticism which can have cascading adverse effects on individuals’ quality of life and relationship satisfaction (Menke *et al.*, 2018). Numerous studies have revealed the distinct role of crus II in ToM/mentalizing processes (Van Overwalle *et al.*, 2020), such as intuitively understanding human actions to promote adaptations to novel situations (Heleven *et al.*, 2019; Van Overwalle *et al.*, 2019) and considering the potential responses of others when guiding goal-oriented behaviour (Van Overwalle and Mariën, 2016; Heleven *et al.*, 2019). The intense experience of shame that is commonly associated with PTSD (Saraiya and Lopez-Castro, 2016) and MI (Litz *et al.*, 2009; Dombo *et al.*, 2013; Kopacz *et al.*, 2015; McEwen *et al.*, 2020) can lead to a profound experience of powerlessness and lack of self-worth (Muris and Meesters, 2014), which may prompt individuals to engage in defensive and avoidant behaviours with the desire to hide or escape from the world. Indeed, according to the results of the RSDI scale, 53.6% of participants in the PTSD group reported experiencing the highest rating of shame (i.e. ‘Very Much So’) after exposure to the MI condition as compared to 6.3% of the control group. Further, none of the participants in the PTSD or control groups reported experiencing feelings of shame after the neutral condition (see Table 2). This adds to extant evidence that individuals with PTSD exposed to MI can elicit strong feelings of shame, which, when coupled with the direct eye gaze, can be overwhelming thus disrupting individuals’ ability to interpret the novel social situation effectively. While neuroimaging studies have revealed connections between the posterior cerebellum and cortical structures central to ToM/mentalizing processes, such as the TPJ, in healthy individuals (Van Overwalle *et al.*, 2015, 2019), future studies should examine these connectivity patterns during gaze processing in PTSD following MI recall.

## Limitations and future directions

Despite the strengths of the study, there are a number of limitations that warrant consideration. First, group sample sizes were small due to the unexpected discontinuation of recruitment with the onset of the Coronavirus disease (COVID-19) pandemic. Future replication of the study with larger samples is needed to confirm the reliability of the findings. Second, we did not distinguish between individuals who met diagnostic criteria for PTSD and those with the PTSD dissociative subtype. Such comparisons may have yielded notable differences in results, as individuals of the dissociative subtype may exhibit over-modulation of higher-order cortical structures manifesting behaviourally as physical and emotional ‘numbing’ (Lanius *et al.*, 2010; Harricharan *et al.*, 2016; Nicholson *et al.*, 2017; Schiavone *et al.*, 2018). Third, our PTSD participants exhibited heterogeneity with regard to trauma histories. While this permits generalizability of the results to the broader population, it could have influenced the salience of neural activation patterns since social cognitive processing may vary depending on the type, onset and duration of the trauma experienced (Nazarov *et al.*, 2014). Relatedly, we did not capture the frequency of exposure to morally injurious events which could have also influenced the current study results. Future studies should focus on comparisons across different etiological profiles of trauma and/or MI to further elucidate the patterns of neural activity during eye gaze processing. Further, as we did not explore the effects of MI independently of PTSD diagnosis, future research may also aim to examine the potential unique effects of MI on direct (*vs* avert) gaze neural processing. Finally, as we only explored activation of neural structures, future studies should conduct network analyses to explore changes in connectivity during exposure to direct *vs* avert gaze post MI event recall.

## Conclusion

Our results demonstrate that social cognitive processing measured during the experience of direct eye gaze after MI recall is altered in individuals with PTSD as compared to healthy controls. These findings reveal that direct eye gaze may instigate compensatory processing in individuals with PTSD, particularly after exposure to a MI event. Significant alterations in the social cognitive processes of individuals with PTSD (e.g. TPJ, right MSFG, left cerebellum crus II) when met with direct eye gaze can have adverse implications for their ability to develop and maintain healthy relationships. The current findings may be useful in informing future interventions aimed at mitigating the effects of PTSD on individuals’ social cognitive functioning.

## Supplementary Material

nsad053_Supp

## Data Availability

The data analysed in the current study are available from the corresponding author (RAL) on reasonable request.
