# Supplementary material for: ‘I am afraid you will see the stain on my soul’: Direct gaze neural processing in individuals with PTSD after moral injury recall
Source: Soc Cogn Affect Neurosci. 2023 Oct 20;18(1):nsad053. doi: 10.1093/scan/nsad053 (PMC10612569; doi:10.1093/scan/nsad053)
Supplement: nsad053_Supp [file nsad053_supp.zip › scan-23-096-File014.docx]

**Supplementary Materials**

**“I am afraid you will see the stain on my soul”: Direct gaze neural processing in individuals with PTSD after moral injury recall**

Krysta Andrews^1,2^, Chantelle S. Lloyd^2,3,4^, Maria Densmore^3,5^, Breanne E. Kearney^6^, Sherain Harricharan^1^, Margaret C. McKinnon^1,2,7^, Jean Théberge^3,5,8^, Rakesh Jetly^9,10^, & Ruth A. Lanius^2,3,5,6*^

^1^ *Department of Psychiatry and Behavioural Neurosciences, McMaster University, Hamilton, ON, Canada*

*^2^ Homewood Research Institute, Guelph, ON, Canada;*

^3^ *Department of Psychiatry, Western University, London, ON, Canada;*

*^4^ Department of Psychology, Neuroscience, and Behaviour, McMaster University, Hamilton, ON, Canada;*

^5^ *Imaging Division, Lawson Health Research Institute, London, ON, Canada;*

^6^ *Department of Neuroscience, Western University, London, ON, Canada;*

^7^ *Mood Disorders Program, St. Joseph’s Healthcare Hamilton, Hamilton, ON, Canada;*

^8^ *Department of Medical Biophysics, Western University, London, ON, Canada;*

^9^ *Canadian Forces, Health Services, Ottawa, ON, Canada;*

*^10^Department of Psychiatry, University of Ottawa, Ottawa, ON, Canada*

***Corresponding Author:**

Ruth A. Lanius, MD, PhD (Ruth.Lanius@lhsc.on.ca)

Address: Western University, University Hospital, 339 Windemere Road, London, ON, Canada N65 5A5

**Moral Injury and Neutral Event-Related Script Information**

**Instructions:**

Before your upcoming visit, we ask that you think about two personal experiences: 1) a moral injury event (see below) and 2) a neutral event that took place around the same time (e.g., brushing teeth, eating breakfast). Neutral events should not have elicited strong positive or negative emotions during the event.

**Definition:**

Moral injury refers to the emotional or spiritual impact of participating in, witnessing, and/or being victimized by actions and behaviours which violate one’s own core moral values and behavioural expectancies of the self and of others.

Moral injury events should elicit strong moral emotions (e.g., shame, guilt, betrayal), somewhere between a 5-8 on a 1-10 scale (with 10 being most severe). We would avoid selecting a moral injury event scoring above 8, since we do not want the memory to be too upsetting when you recall it.

**Other Moral Injury Definitions:**

- A disruption in an individual’s confidence and expectations about one’s own or others’ motivation or capacity to behave in a just and ethical manner.^1^
- An inability to justify personal actions or witnessed events, leading to these events having an unsuccessful integration into pre-existing moral schemas.^2^
- A deep soul wound that pierces a person’s identity, sense of morality, and relationship to society.^3^

**References**

1. Drescher, K.D., Foy, D.W., Kelly, C., Leshner, A., Schutz, K., & Litz, B. (2011). An exploration of the viability and usefulness of the construct of moral injury in war veterans. *Traumatology, 17*(1), 8-13.
2. Litz, B. T., Stein, N., Delaney, E., Lebowitz, L., Nash, W. P., Silva, C., & Maguen, S. (2009). Moral injury and moral repair in war veterans: A preliminary model and intervention strategy. *Clinical psychology review*, *29*(8), 695-706.
3. Silver, M. (2011). Our morality: A defense of moral objectivism. *Philosophy Now*, *83*, 21-24.
