# Supplementary figures and images for: ‘I am afraid you will see the stain on my soul’: Direct gaze neural processing in individuals with PTSD after moral injury recall
Source: Soc Cogn Affect Neurosci. 2023 Oct 20;18(1):nsad053. doi: 10.1093/scan/nsad053 (PMC10612569; doi:10.1093/scan/nsad053)

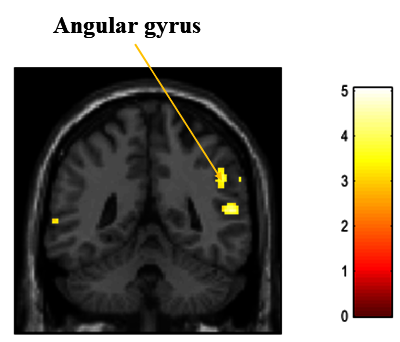

Supplement: nsad053_Supp [file nsad053_supp.zip › scan-23-096-File013.tif]
